# Editorial: Ethylene: a key regulatory molecule in plants, Volume II

**DOI:** 10.3389/fpls.2023.1222462

**Published:** 2023-06-16

**Authors:** Nafees A. Khan, Antonio Ferrante, M. Iqbal R. Khan, Peter Poor

**Affiliations:** ^1^ Department of Botany, Aligarh Muslim University, Aligarh, India; ^2^ Department of Agricultural and Environmental Sciences, Università degli Studi di Milano, Milan, Italy; ^3^ Department of Botany, Jamia Hamdard, New Delhi, India; ^4^ Department of Plant Biology, University of Szeged, Szeged, Hungary

**Keywords:** ethylene, physiology, metabolism, phytohormones, signaling molecules

Ethylene (ET) is a gaseous hormone that regulates plant developmental processes and tolerance to biotic and abiotic stresses (Abeles et al., 1992; Chen et al., 2022; Fatma et al., 2022). The use of ET for crop management and genetic enhancement has substantially expanded with advancements in our understanding of functional genomics. This article collection consists of two reviews and five original research articles that provide scientific references, valuable clues, and molecular and transcriptomic strategies for coping with a range of crop disasters such as metal toxicity, nutrient deficiency, and oxidative imbalance not only in angiosperms but also in liverworts.

The biosynthesis of this plant hormone starts with methionine that through the activities of specific enzymes leads to ET production. The two key enzymes are the 1-aminocyclopropane-1-carboxylate synthase (ACS), which is encoded by *ACS* genes, and 1-aminocyclopropane-1-carboxylate oxidase (ACO), encoded by *ACO* genes. The 1-aminocyclopropane-1-carboxylate (ACC) is considered the direct precursor of ET (Sauter et al., 2013). The linear signaling pathway is as follows:

ET—||ET receptors → CTR1—||EIN2 → EIN3/EILs → ERFs → ET responses

The biological function of ET depends on the CONSTITUTIVE TRIPLE RESPONSE1 (CTR1*)*, which is a kinase, the ETHYLENE INSENSITIVE2 (EIN2) that is a protein located in the endoplasmic reticulum (ER) membrane, EIN3, ETHYLENE INSENSITIVE LIKEs (EILs), and ETHYLENE RESPONSE FACTORs (ERFs) that are transcription factors (Binder, 2020). In the absence of ET, CTR1 phosphorylates EIN2, preventing the cleavage and translocation of the C-terminal end (CEND) of EIN2 into the nucleus. In the presence of ET, CTR1 is inactivated, resulting in the dephosphorylation of EIN2 and its cleavage. CEND is then translocated into the nucleus, enhancing EIN3/EIL1 binding to target genes and promoting transcriptional changes (Binder, 2020; Angulo et al., 2021).

## ET-mediated abiotic stress regulation in angiosperms and liverworts

Industrial runoff contains heavy metals, contaminating soil and poisoning crops. Sehar et al. investigated the role of ET in overcoming arsenic (As) phytotoxicity in the presence of selenium in mustard. The higher accumulation of As in leaves than in roots was the primary cause of reduced photosynthetic performance and reduced growth in mustard. ET application alone and in the presence of Se resulted in adaptive responses to As toxicity through increased expression of *ASCORBATE PEROXIDASE (APX)* and *GLUTATHIONE REDUCTASE (GR)*, accumulation of reduced glutathione (GSH) and the suppression of abscisic acid (ABA)-mediated stomatal closure. Evolutionary insights into ET action were presented by Bharadwaj et al. The mechanism of ET action in liverwort (*M. polymorpha*) in response to a range of abiotic stresses was found to be markedly similar to that of angiosperms against a range of abiotic stresses. Mutant lines *Mpein3* and *Mpctr1* defective in positive and negative regulators of ET were compared with respective wild types against a range of abiotic stresses such as heat, salinity, nutrient deficiency, and far-red light. According to a previous study (Li et al., 2020), lines defective in the EIN3 transcription factor would be insensitive to ET response, indicating that EIN3 is a positive regulator of ET signaling, whereas the *CTR1* mutants would display constitutive ET responses, suggesting that it is a negative regulator of ET perception. As hypothesized, *Mpctr1* mutant lines showed prominent ET response and more resilience under sublethal conditions such as 29°C, 10 mM NaCl, 1/20 Gamborg’s dilution, or 50 days of exposure to far-red light, whereas *Mpein3* mutant showed lower resilience than wild type.

## Strategic control over nutrient deficiency and post-harvest ripening

Two comprehensive reviews dealing with TARGET OF RAPAMYCIN (TOR) and ET interconnection under nutrient deficiency and strategic post-harvest processing for increasing the shelf life of fruits and vegetables showcased detailed reports on the ET action and control mechanisms. Garcia et al. set ground on the so-called “stop growing” and “searching for nutrient” schemes to deal with nutrient deficit through TOR and ET interaction. Authors described reports where TOR and ET share antagonistic relationships: TOR blocks ET signaling alongside CTR1 by phosphorylating EIN2 and EIN3. However, nutrient deprivation inactivates TOR reactivating ET signaling.

In their review of post-harvest practices, Cocetta and Natalini describe how apart from conventional means such as chilling treatments and antisense approaches for *ACS* and *POLYGALACTURONASE* (*PG)* genes, transgenic approaches have been applied to elucidate ET biochemical and metabolic pathways. Ripening-related transcription factors of the APETALA2/ETHYLENE-RESPONSE FACTOR **(**AP2/ERF) family have been studied in tomatoes for their role in regulating ripening-responsive genes (Fenn and Giovannoni, 2021). Genetic analysis has led to the identification of ripening mutants like *ripening inhibitor (rin), non-ripening (nor), colorless non-ripening (Cnr), green-ripe (Gr), green flesh (gf), high pigmnet1 (hp1), high pigment2* (*hp2), and never ripe (Nr)* in tomato (Osorio et al., 2020). These mutants are used commercially to develop new hybrids with longer shelf life. The CRISPR/Cas9 approach is promising, and it has been applied to regulatory protein genes *CNR* and *NOR* and to transcription factors *AP2a, FUL1*, and *FUL2* to achieve targeted deletion or substitution (Wang et al., 2019).

## ACC-DI peptide (di-ACC) and AP2/ERFs regulating ET responses

Vaughan-Hirsch et al. investigated the biological function of custom-synthesized 1- aminocyclopropane-1-carboxylic-acid dipeptide (di-ACC) molecule taken up by Arabidopsis just as ACC, in part via LYSINE HISTIDINE TRANSPORTERS (e.g., LHT1). Once taken up, the ACC dimer can evoke a triple response phenotype in dark-grown seedlings, reminiscent of ET responses induced by ACC itself, albeit less efficiently than ACC. Moreover, di-ACC operates via the known ET signaling pathway and not through ACC. Altogether, the study supported that di-ACC appears to be transported and processed into ET like ACC, and it can increase ET production levels and trigger further ET reactions in Arabidopsis.

Considering the importance of AP2/ERF in resilience responses, Ding et al. conducted a genome-based study in barley to explore the role of AP2/ERFs in starch synthesis. After re-examining the available genome database (Morex), these researchers identified 64 new genes in the *HvAP2/ERF* gene family and corrected some previously misannotated and duplicated genes. *HvAP2-18* was identified as a promising candidate gene to shed light on the mechanism governing the production of starch in barley.

The role of the *AP2/ERF* gene family in resistance of *N. benthamiana* against *P. infestans* infection after INF1 (elicitin from *P. infestans*) treatment has been confirmed by Imano et al. The authors successfully demonstrated that *NbERF-IX-33* transcription factors are responsible for the accumulation of the sesquiterpenoid phytoalexin capsidiol that serves as an important protective compound against *P. infestans* infection.

**Figure 1:**
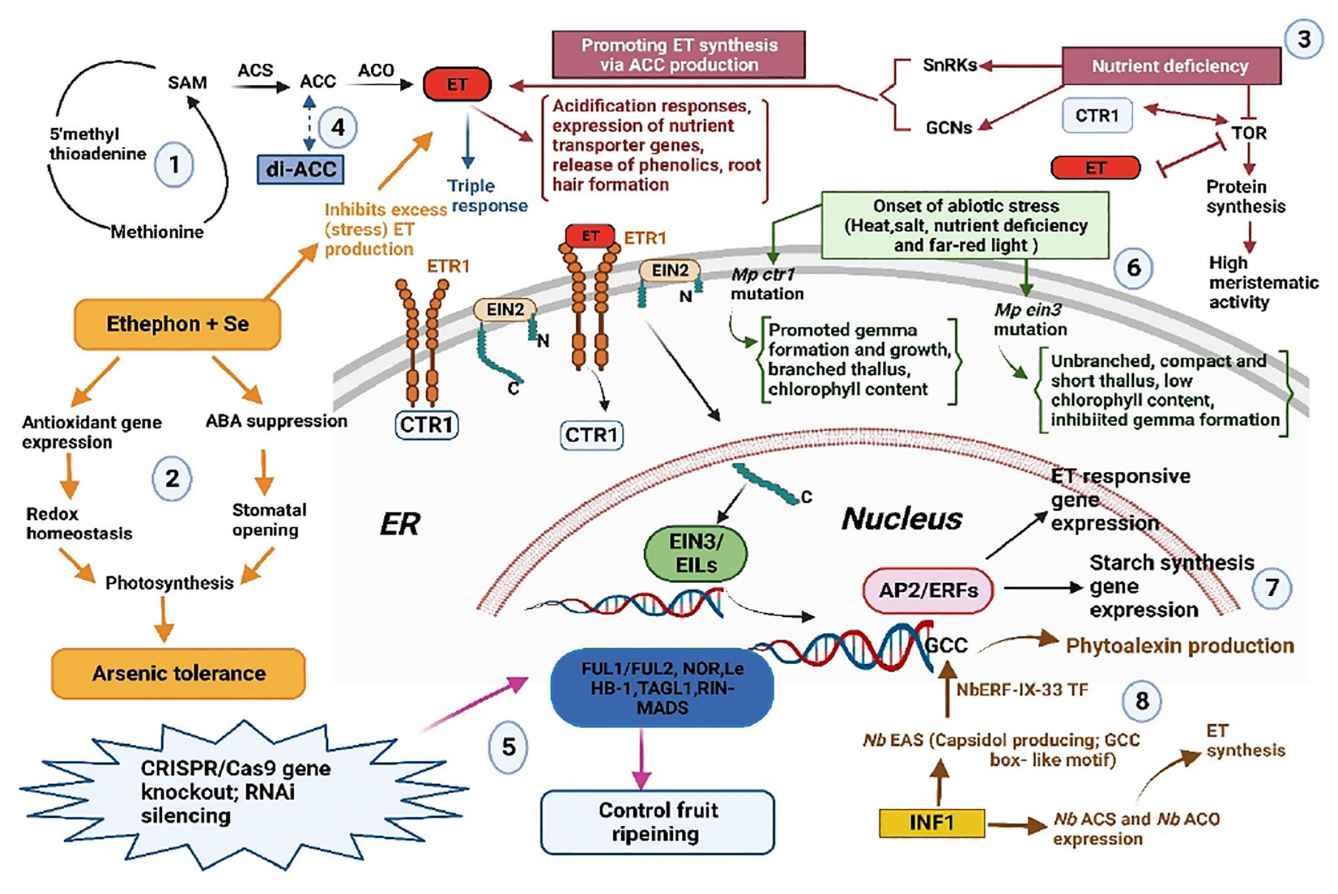
Summary of the articles published in the volume. (1) ET biosynthesis and perception leading to various ET-related responses; (2) Findings of Zebus et al. on Sc, ET and ABA interaction for As tolerance; (3) Garcia et al. report on inter-relationship between ET and TOR system; (4) Vanughan-Hirsch et al. report on di-ACC action; (5) Cocetta & Natalini review on fruit ripening; (6) Bharadawaj et al. report on *Marchantia polymorpha* mutants *Mp ein3 and Mp ctrl;* (7) Ding et al. finding on the role of AP2/ ERFS in starch synthesis; and (8) Finding of Imano et al. on phytoalexin production against *Phytophthora infestans* through *NbERF-IX-33* in Nicotiana benthamiana. Arrows indicate 
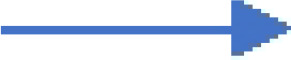
 promotion; 
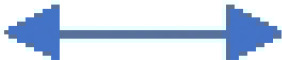
 Similarity in function; 
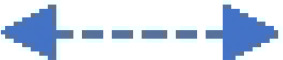
 similarity but less substrate affinity than ACC; 
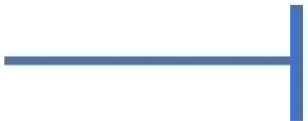
 inhibition; 
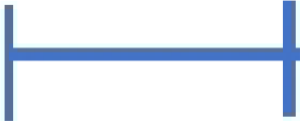
 antagonistic action; ABA, Abscisic acid; ACO, ACC oxidase; ACC, 1-aminocyclopropane-1-carboxylic acid, ACS, ACC synthase; AP2/ERFs, APETALA2/ETHYLENE RESPONSE FACTOR, As, arsenic, CTR1, CONSTITUTIVE TRIPLE RESPONSE; di-ACC, 1- aminocyclopropane-1-carboxylic acid-dipeptide; EIN, ETHYLENE INSENSITIVE; EIL, EIN3-LIKE, ET, ethylene, ETR, ETHYLENE RESPONSE, ER, Endoplasmic reticulum; FUL1/FUL2; NOR; Le HB-1,TAGL.RIN-MADS, Genes for fruit ripening; GCN, GENERAL CONTROL NON-DEREPRESSIBLE; SAM, S- adenosyl-l-methionine, Se, Selenium; SnRKS, SUCROSE NON-FERMENTING1- RELATED PROTEIN KINASEA; INF, Elicitin from *Phytophthora infestans*.

The articles in this collection contribute to our understanding of ET as a regulatory signal in plants. ET is again demonstrated to serve as an important mediator of stress in liverworts and angiosperms. Future studies in this exciting area will help to unravel stress response networks and how they interact with ET to determine which pathways may have existed in the ancestor of early land plants and to create strategies for reducing the adverse effects of abiotic stresses, which are becoming more common as a result of climate change. A graphical summary of the articles in the volume is presented in **Figure 1**.

## Author contributions

All authors listed have made a substantial, direct, and intellectual contribution to the work and approved it for publication.
